# In vivo modulation of the behavioral effects of nicotine by the coumarins xanthotoxin, bergapten, and umbelliferone

**DOI:** 10.1007/s00213-016-4279-9

**Published:** 2016-04-14

**Authors:** Barbara Budzynska, Krystyna Skalicka-Wozniak, Marta Kruk-Slomka, Malgorzata Wydrzynska-Kuzma, Grazyna Biala

**Affiliations:** Department of Pharmacology and Pharmacodynamics, Medical University of Lublin, Lublin, Poland; Department of Pharmacognosy with Medicinal Plants Unit, Medical University of Lublin, Lublin, Poland

**Keywords:** Coumarins, Nicotine, Memory, Depression, Mice

## Abstract

**Rationale:**

Nicotine, a dominant alkaloid found in tobacco, is responsible for physical dependence, as well as addiction to cigarette smoking; consequently, smoking cessation is a very difficult process. Hepatic cytochrome P-450 2A6 (CYP2A6) is involved in the 70–80 % of the initial metabolism of nicotine and its co-metabolites. As this metabolism is slowed by inhibitors of CYP2A6, this kind of enzymatic inhibition has been proposed as a novel target for smoking cessation.

**Objectives:**

Nicotine administered alone improved memory acquisition and consolidation as well as exerted antidepressive activity in animal models. These effects persist for 24 h. However, they are completely extinguished 48 h after administration.

**Methods:**

To investigate if the coumarins prolong the behavioral effects of nicotine, the forced swimming test (FST)—animal models of depression, and passive avoidance (PA) test—memory and learning paradigm were used.

**Results:**

This study revealed that three CYP2A6 inhibitors: two furanocoumarins, xanthotoxin (15 mg/kg) and bergapten (25 mg/kg), and the simple coumarin umbelliferone (25 mg/kg), prolonged the antidepressive and procognitive effects of nicotine.

**Conclusions:**

These natural products may offer a new approach to the treatment of nicotinism as antidepressant and memory improvement actions are one of the main factors of nicotine dependence.

## Introduction

Nicotine, the main component of tobacco smoke, influences the mood and emotional tension, as well as contributes to development of physical and mental dependence. It is well documented that nicotine exerts cognitive effects (Herman et al. 2010; Levin 2002; Levin et al. 2009), analgesia (Marubio et al. 1999), and an influence on anxiety- (File et al. 2000) or depression-like behaviors (Hayase 2011). The effects of nicotine have been extensively investigated not only in humans but also in animals and several cell systems (Biala et. al. 2014; Budzynska et al. 2012b; Dani and De Biasi 2001; Kruk-Slomka et al. 2012; Malin 2001). Moreover, nicotine has strong addictive potential resulting from activation of dopaminergic neurons in the mesolimbic system, which are part of the reward system. Thus, quitting smoking is very challenging and nicotine replacement therapy (NRT), with products that supply low doses of nicotine, may be helpful in this process (Benowitz 2009).

Coumarins, based on the 2*H*-1-benzopyran-2-one scaffold, are widely distributed in the plant kingdom and occur in the seeds, roots, and leaves of many plant species, e.g., *Apiaceae*, *Rutaceae*, *Fabaceae*, *Orchidaceae* (O’Kennedy and Thornes 1997 Coumarins possess a range of diverse pharmacological properties. Plants containing coumarins are used in traditional medicine all over the world for their antiproliferative, anticonvulsant, anxiolytic, and procognitive effects (Abed et al. 2001; Budzynska et al. 2012a; Garcia-Argaez et al. 2000; Kawaii et al. 2001; Luszczki et al. 2007).

Furthermore, several enzymes are involved in the metabolism of nicotine. In humans, nicotine is metabolized to the extent of 70–80 % to cotinine (5-oxo-nicotine) in a biphasic process. The first step is catalyzed by hepatic cytochrome P-450 2A6 (CYP2A6) to produce the nicotine iminium ion. The next step is catalyzed by an aldehyde oxidase to produce cotinine (Hukkanen et al. 2005; Murphy et al. 2005). CYP2A6 also converts cotinine to *trans*-3-hydroxycotinine. All three products, e.g., nicotine, cotinine, and *trans*-3-hydroxycotinine, are glucuronidated and eliminated in the urine (Yamanaka et al. 2004). Therefore, inhibition of the hepatic metabolism of nicotine results in the slower elimination of nicotine in smokers (Sellers et al. 2003) and increases the pharmacological effects of nicotine in animals (Alsharari et al. 2014; Damaj et al. 2007).

In addition to nicotine, coumarins are important substrates for human CYP2A6 and CYP2A13 (Raunio and Rahnasto-Rilla 2012; Zhang et al. 2001). More significantly, it was shown that these compounds inhibit CYP2A5-mediated nicotine metabolism in vivo in the mice (Damaj et al. 2007). CYP2A5 is a mouse cytochrome P450 monooxygenase and it shows a high degree of similarity with human CYP2A6 and CYP2A13 in protein sequence and substrate specificity (Su and Ding 2004).

Xanthotoxin is an inhibitor of CYP2A5, monoamine oxidase (MAO), acetylcholinesterase (AChE), and butyrylcholinesterase (BChE) (Skalicka-Wozniak et al. 2014). Bergapten, an analog of xanthotoxin, is the most potent inhibitor of CYP3A4 (Ho et al. 2001). Furthermore, bergapten inhibits the activity of BChE and AChE, which results in an increase of acetylcholine (ACh) and butyrylcholine (BCh) in the brain (Senol et al. 2011), and bergapten inhibits the activity of MAO and shows antidepressant effects (Huong et al. 1999). Umbelliferone also has the ability to inhibit MAO (Seon et al. 2006). The data suggest that this coumarin interacts with the benzodiazepine binding site of the GABA-A receptor, which may indicate anxiolytic activity (Singhuber et al. 2011). Nicotine metabolism is slowed by hepatic enzyme inhibitors, and since CYP2A6 is responsible for 70–80 % of the initial metabolism of nicotine (Al Koudsi et al. 2010), CYP2A6 inhibition has been proposed to be a novel target for smoking cessation. Consequently, these natural products may offer a new approach in the treatment of nicotinism by prolonging the effect of low levels of nicotine contained in NRT. The potential problem of lack of a sustained effect of nicotine with NRTs does not apply to the nicotine skin patch, which delivers 24-h continuous dosing of nicotine. However, it may be an issue with acute dosing forms, such as nicotine gum or lozenge, which are typically underutilized by patients. Two furanocoumarins, xanthotoxin and bergapten, and the simple coumarin, umbelliferone, were chosen for the present study to investigate if they prolong the behavioral effects of nicotine in animal models of depression, as well as memory and learning.

## Animals and methods

### Animals

The experiments were carried out on 2-month old, naïve male Swiss albino mice (Farm of Laboratory Animals, Warsaw, Poland). At the beginning of the experiments, they weighed 20–25 g. The mice were maintained under standard laboratory conditions (12 h light/dark cycles, room temperature 21 ± 1°C). The mice were kept in colony cages (8–10 per cage) with free access to food (Agropol, Poland) and tap water. After 1 week of adaptation to laboratory conditions, the animals were randomly assigned to experimental group. Each group consisted of 8–10 mice. All experiments were performed between 08:00 and 15:00 hour. Each mouse was used only once.

All experiments were conducted according to the National Institute of Health Guidelines for the Care and Use of Laboratory Animals (8th edition) and to the European Community Council Directive for the Care and Use of Laboratory Animals of 22 September 2010 (2010/63/*EU*) and were approved by the local ethics committee (44/2015).

### Drugs

The following compounds were tested: (–)-nicotine hydrogen tartrate (expressed as a salt, 0.1 and 0.2 mg/kg, Sigma–Aldrich, St. Louis, MO, USA), xanthotoxin [8-methoxyfuro[3,2-*g*]chromen-7-one] (15 mg/kg), bergapten [5-methoxyfuro[3,2-*g*]chromen-7-one] (25 mg/kg), and umbelliferone [7-hydroxychromen-2-one] (25 mg/kg). Coumarins were isolated as described below. Nicotine was dissolved in saline solution (0.9 % NaCl) and administered subcutaneously (s.c.) at a volume of 10 mL/kg. The pH of the nicotine solution was adjusted to 7.0. Coumarins were suspended in a 1 % solution of Tween 80 (Sigma-Aldrich, St. Louis, MO, USA) dissolved in a saline solution and administered intraperitoneally (i.p.) at the volume of 10 mL/kg. Fresh drug solutions were prepared on each day of experimentation. Control groups received saline injections of the same volume and through the same route of administration.

### Isolation of coumarins

Xanthotoxin was isolated from the dichloromethane extract of the fruits of *Pastinaca sativa* L. (Apiaceae) (Skalicka-Wozniak et al. 2014), while bergapten and umbelliferone were obtained from the dichloromethane and methanol extracts of the fruits of *Heracleum leskovii* Grossch. (Apiaceae), respectively (Kielbus et al. 2013; Skalicka-Wozniak et al. 2015). All plants were obtained from the Medicinal Plant Garden, Department of Pharmacognosy, Medical University of Lublin, Poland, in the summer of 2010.

The extracts were separated using a high-performance counter-current chromatograph Spectrum (Dynamic Extractions, Slough, UK) and semipreparative coil with a capacity of 137 mL was used. A solvent system composed of heptane–ethyl acetate–methanol–water (1:1:1:1 *v*/*v*) was used for the separation of xanthotoxin, (6:5:6:5) for bergapten isolation, and (1:2:1:2) for umbelliferone. The upper phase was used consistently as the stationary phase, the rotation was set at 1600 rpm, and the mobile phase was pumped into the column at a flow rate of 6.0 mL/min, and the effluent from the coil was monitored at 254 nm. Identification of the eluted compounds was confirmed by HPLC-DAD and LC-TOF-MS analyses. The purity of isolated compounds was higher than 98 %.

### The passive avoidance task

The experimental apparatus consisted of two acrylic compartments:

- Lighted compartment (10 × 13 × 15 cm); illuminated by a fluorescent light (8 W).
- Darkened compartment (25 × 20 × 15 cm); equipped with an electric floor Venault et al. 1986).

On the first day of the experiment (pretest), each mouse was placed in the light chamber and allowed to move freely in it for 30 s. After this time, the guillotine door between the compartments was raised to allow the mice to enter the dark chamber. When the experimental animal entered the dark compartment, the guillotine door was closed and electric foot-shock (0.15 mA) of 2-s duration was delivered to the mouse through the grid floor. The latency time (TL1) was recorded for entering the dark chamber. When the mouse failed to enter the dark chamber within 300 s, it was placed into this box. The guillotine doors were closed and an electric foot-shock (0.15 mA) of 2-s duration was delivered to the mouse through the grid floor. The TL1 was recorded as 300 s.

Twenty-four hours later, the same mouse was placed individually in the light chamber. After 30 s, the guillotine door was raised to allow the mouse to enter the dark chamber. The latency time (TL2) was recorded for reentering the dark chamber. In this trial, no foot-shock was applied. When the mouse failed to enter the dark chamber within 300 s, the TL2 was recorded as 300 s (Allami et al. 2011; Borowicz et al. 1995; Hiramatsu et al. 1998; Javadi-Paydar et al. 2012). The experiment involves an examination of different memory stages:

– Acquisition = formation of memory traces (the animal received substances prior to the test)

– Consolidation (the animal received substances after the test)

### Forced swimming test

The forced swimming test (FST) is used for assessing antidepressant activity (Lucki 1997). The experimental animals were immersed individually in clear glass cylinders (diameter 10 cm; height 25 cm) containing 10 cm of water (to prevent their tails from touching the bottom) at a temperature of 23–25 °C. The animals were allowed to swim without any possibility of escape. After 2 min of observing the mouse in the water, the test was started. Immobility was manually recorded over the 4-min testing period. Animal mobility is limited apart from the movements necessary to keep the head above the water. After 6 min of test session, the animal was removed, dried, and returned to their cage. Antidepressant activity was characterized by a decrease in the immobility time.

### Locomotor activity

The locomotor activity of mice was measured using photoresistor actimeters (circular cage, diameter 25 cm, two light beams). The animals were placed individually in an actimeter for 60 min. When mice crossed the light beams, it was recorded as the locomotor activity. Locomotor activity of mice was measured with photoresistor actimeters (circular cages, diameter 25 cm, two light beams).

## Treatment

**Diagram 1:**
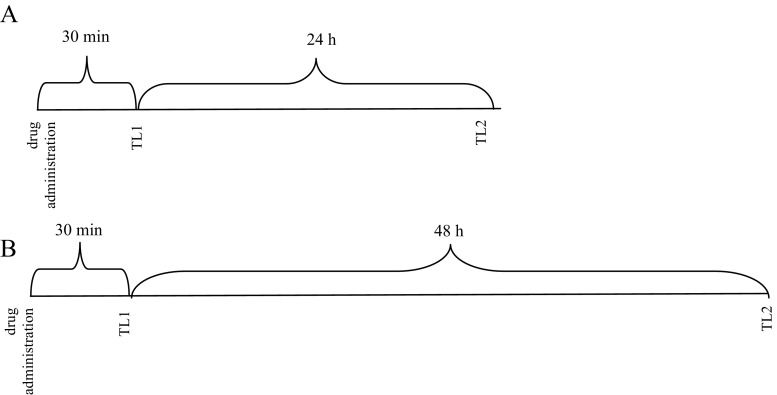
Diagram shows the schedule of administration of coumarins (i.p.) and nicotine (s.c.) in the PA paradigms. The tests were performed 30 min after drug administration (TL1) and 24 h later (TL2) to observe acute effects of coumarins and nicotine on memory acquisition processes (**a**) or 30 min after drug administration (TL1) and then 48 h later (TL2) to observe if coumarins prolonged effects of nicotine on memory acquisition processes (**b**)

**Diagram 2:**
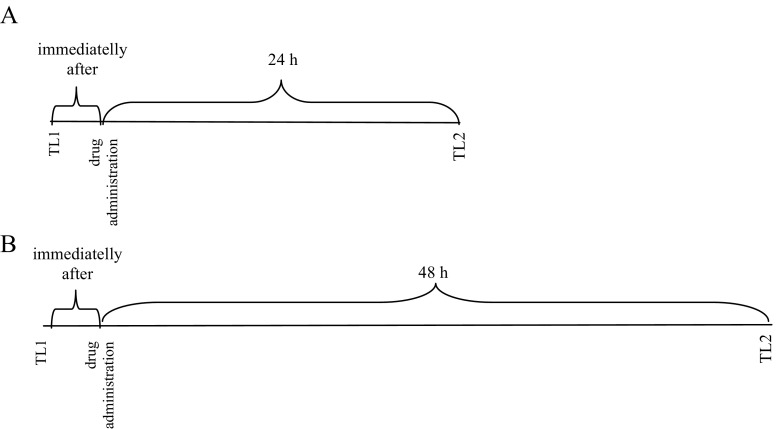
Diagram shows the schedule of administration of coumarins (i.p.) and nicotine (s.c.) in the PA paradigms. To observe acute effects of coumarins and nicotine on memory consolidation processes, **a** the drugs were administered immediately after TL1 trial and 24 h later, the TL2 trial was performed. In turn, to observe if coumarins prolonged effects of nicotine on memory consolidation processes, **b** the drugs were administered immediately after TL1 trial and 48 h hours later, the TL 2 trial was performed

**Diagram 3:**
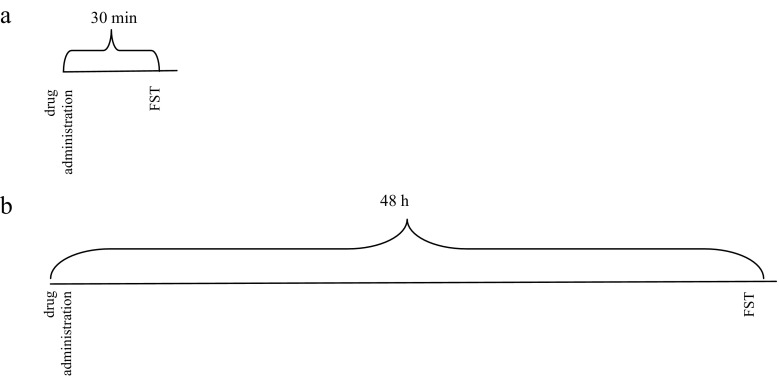
Diagram shows the schedule of administration of coumarins (i.p.) and nicotine (s.c.) in the FST. The test was performed 30 min after drug administration to observe acute effects of coumarins and nicotine (**a**) or 48 h after drug administration to observe if coumarins prolonged the effects of nicotine (**b**)

**Fig. 1:**
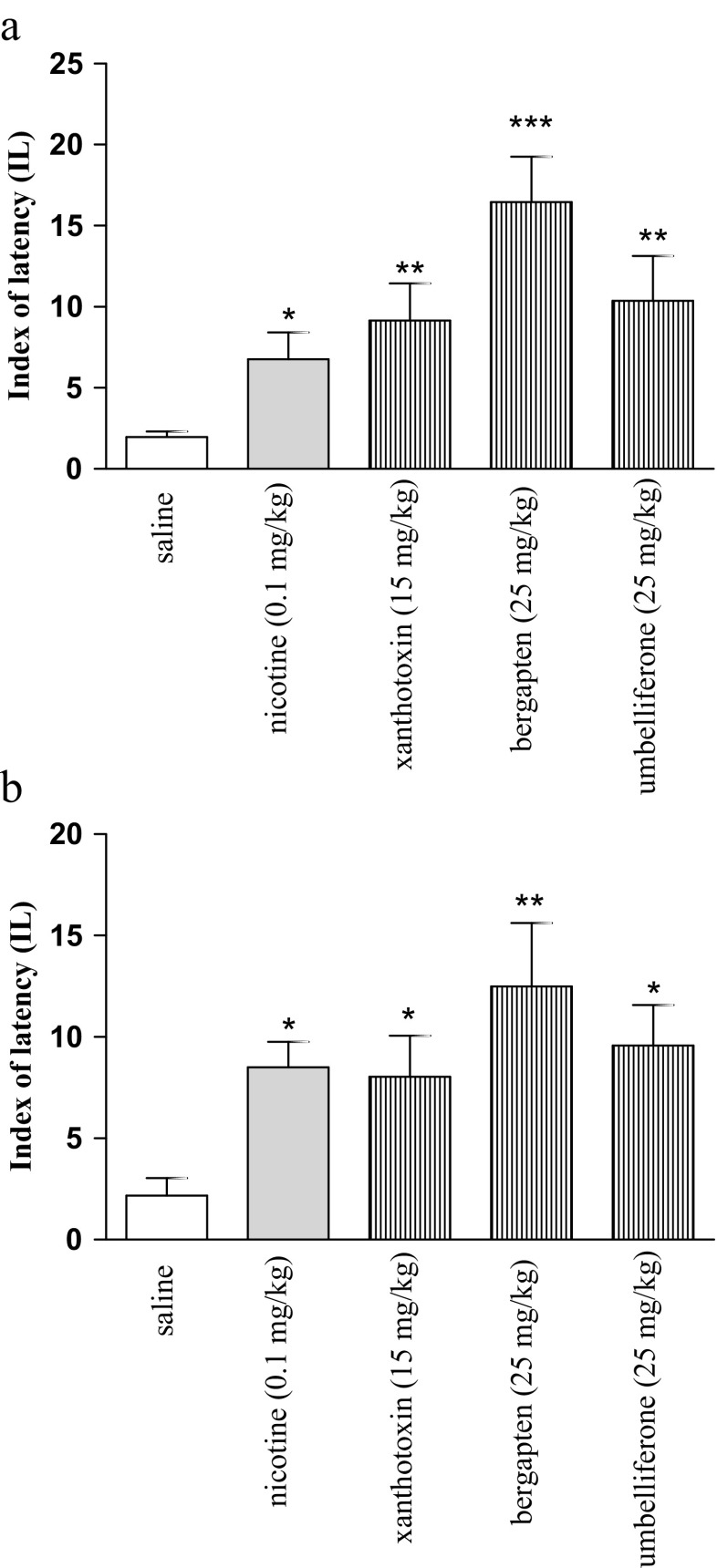
Effects of nicotine (0.1 mg/kg), xanthotoxin (15 mg/kg), bergapten (25 mg/kg), umbelliferone (25 mg/kg), or saline injection on memory acquisition trial (**a**) and consolidation trial (**b**) using the PA test in mice. Drugs were administered 30 min before the first trial (**a**) or immediately after the first trial (**b**), and animals were retested 24 h after the injection; *n* = 8–10; the means ± SEM; *p < 0.05; **p < 0.01 ***p < 0.001 vs. saline-treated control group; Tukey’s test

The doses of the coumarins and nicotine as well as time of the experiments were chosen based on literature data (Alsharari et al. 2014; Bagdas et al. 2014), a recently published article from this laboratory (Budzynska et al. 2013), and preliminary studies. To determine the effects of nicotine (0.1 mg/kg), xanthotoxin (15 mg/kg), bergapten (25 mg/kg), and umbelliferone (25 mg/kg) on memory acquisition, trial drugs were administered 30 min before the first trial (TL1) and animals were retested 24 h after the injection (TL 2) (Diagram 1a). To determine the effects of drugs on memory consolidation trial, the compounds were administered immediately after the first trial (TL1) and animals were retested 24 h after the injection (TL 2) (Diagram 2a). Moreover, to assess the effects of nicotine (0.2 mg/kg), xanthotoxin (15 mg/kg), bergapten (25 mg/kg), umbelliferone (25 mg/kg), or saline injection on depressive-like behavior, drugs were administered 30 min before the FST (Diagram 3a) (Fig. 1).

**Fig. 2:**
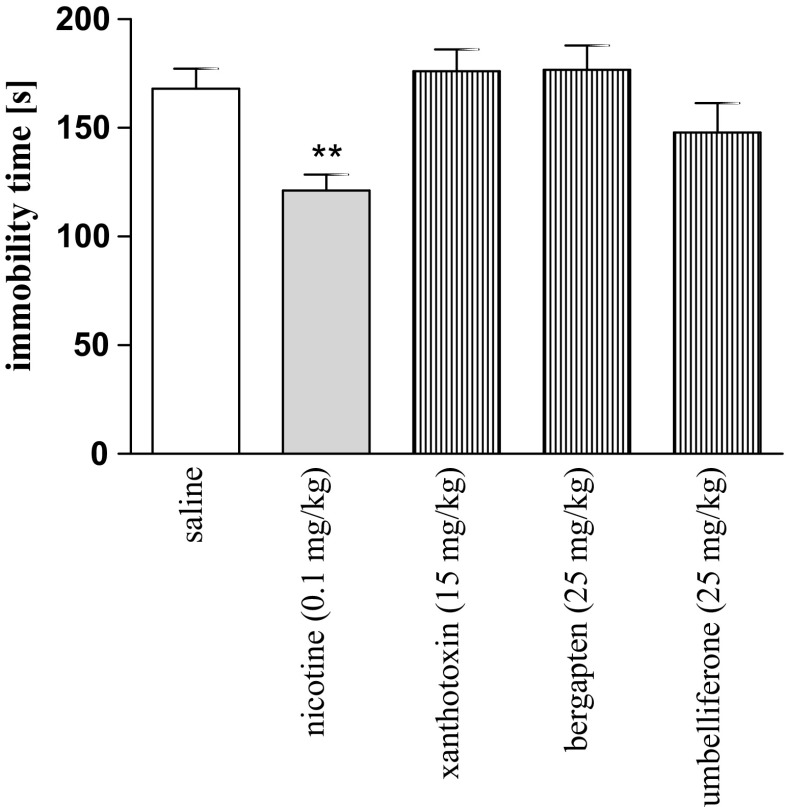
Effects of nicotine (0.2 mg/kg), xanthotoxin (15 mg/kg), bergapten (25 mg/kg), and umbelliferone (25 mg/kg) or saline injection on the total duration of immobility in the FST in mice. Drugs or saline were administered 30 min before the test and animals were retested 24 h after the injection; *n* = 8–10; the means ± SEM; *p < 005 vs. saline control group; Tukey’s test

To determine if coumarins prolong the behavioral effects of nicotine in animal models of depression, as well as memory, and learning the animals were allocated to the following drug groups: nicotine (0.1 or 0.2 mg/kg, s.c.) + saline, saline + xanthotoxin (15 mg/kg, i.p.), saline + bergapten (25 mg/kg, i.p.), or saline + umbelliferone (25 mg/kg, i.p.), and nicotine (0.1 or 0.2 mg/kg, s.c.), coadministered with xanthotoxin (15 mg/kg, i.p.), bergapten (25 mg/kg, i.p.), or umbelliferone (25 mg/kg, i.p.). The TL1 trial in the PA tests was performed 30 min after drug administration (memory acquisition) or immediately before drug administration (memory consolidation) and then the TL2 trial was performed 48 h later (Diagrams 1b and 2b). The FST was made 48 h after drugs administration (Diagram 3b).

### Locomotor activity

In order to measure locomotor effects of nicotine (0.1 and 0.2 mg/kg), xanthotoxin (15 mg/kg), bergapten (25 mg/kg), or umbelliferone (25 mg/kg), animals were injected with drugs and immediately placed in the activity chamber. Locomotor activity, i.e., the number of photocell beam breaks was automatically recorded.

## Statistical analysis

Data were analyzed using two-tailed ANOVA test with post hoc Tukey’s test. All data are shown as means (±SEM). The results of *p* < 0.05 were considered as statistically significant.

For the memory-related behaviors, changes in the PA performance were expressed as the difference between the retention and training latencies and were taken as an index of latency (IL). IL was calculated for each animal and reported as the ratio:

IL = TL2-TL1/TL1

TL1—the time taken to enter the dark compartment during the training

TL2—the time taken to reenter the dark compartment during the retention (Budzynska et al. 2015; Chimakurthy and Talasila 2010).

## Results

### Single injection of nicotine, xanthotoxin, bergapten, and umbelliferone affects memory-related processes 24 h after drug administration in the PA test in mice

One-way ANOVA revealed that, at the acquisition trial, the acute administration of nicotine (0.1 mg/kg), xanthotoxin (15 mg/kg), bergapten (25 mg/kg), and umbelliferone (25 mg/kg) significantly changed IL values [F(4,43) = 4.56; *p* = 0.0040]. The post hoc Tukey’s test showed that nicotine (*p* < 0.05), xanthotoxin (*p* < 0.01), bergapten (*p* < 0.001), and umbelliferone (*p* < 0.01) significantly increased IL as compared to the saline-treated mice, indicating improvement of memory and learning processes as measured 24 h after their administration (Fig. 1a).

One-way ANOVA revealed that, at the consolidation trial, the acute administration of nicotine (0.1 mg/kg), xanthotoxin (15 mg/kg), bergapten (25 mg/kg), and umbelliferone (25 mg/kg) significantly changed IL values [F(4,41) = 2.84; *p* = 0.0378]. The post hoc Tukey’s test showed that nicotine (*p* < 0.05), xanthotoxin (*p* < 0.05), bergapten (*p* < 0.01), and umbelliferone (*p* < 0.05) significantly increased IL as compared to the saline-treated mice, indicating improvement of memory and learning processes (Fig. 1b).

### Effects of single injection of nicotine, xanthotoxin, bergapten, and umbelliferone on depression-like behaviors 24 h after drug administration in the FST in mice

Figure 2 shows the effect of nicotine (0.2 mg/kg), xanthotoxin (15 mg/kg), bergapten (25 mg/kg), and umbelliferone (25 mg/kg) on depression-like behaviors in the FST (one-way ANOVA: F(4,47) = 7.18, *p* = 0.0002). A post hoc Tukey’s analysis showed significant changes only after nicotine administration at the doses of 0.2 mg/kg in the immobility time as compared with the saline-treated mice (*p* < 0.01) as measured 24 h after its administration (Fig. 2).

### Single injection of xanthotoxin, bergapten, and umbelliferone affects memory-related processes induced by nicotine 48 h after drug administration in the PA test in mice

**Fig. 3:**
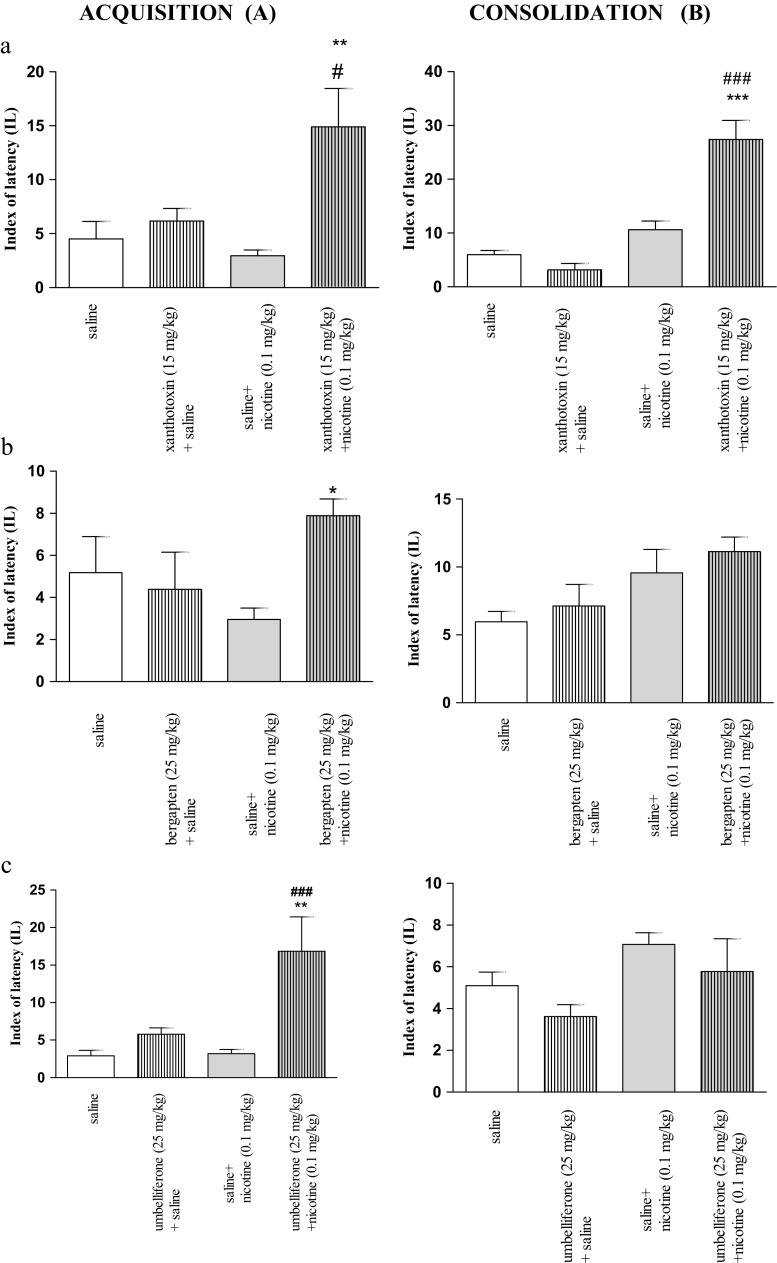
Effects of the injection of coumarins on nicotine-induced memory acquisition trial (**a**) and consolidation trial (**b**) after extinction using the PA test in mice. Xanthotoxin (15 mg/kg; *a*), bergapten (25 mg/kg; *b*), and umbelliferone (25 mg/kg; *c*), saline and/or nicotine (0.1 mg/kg) were administered 30 min before the first trial (**a**) or immediately after the first trial (**b**), and animals were retested 48 h after the last injection; *n* = 8–10; the means ± SEM; *p < 0.05; **p < 0.01 ***p < 0.001 vs. nicotine-treated control group, #*p* < 0.05, ###*p* < 0.001 vs. xanthotoxin- or umbelliferone-treated control group; Tukey’s test

Figure 3a (a) indicates the effects of the injection of xanthotoxin (15 mg/kg) and nicotine (0.1 mg/kg, s.c.) alone or in combination on memory acquisition 48 h after drug administration in the PA task (two-way ANOVA: pretreatment [F(1,28) = 10.91, *p* = 0.0026], interactions [F(1,28) = 6.20, *p* = 0.0190] without treatment effect [F(1,28)=3.02, *p* = 0.0934]). In the xanthotoxin and nicotine-treated groups, there were no significant changes in the IL value as compared with the saline-treated mice (*p* > 0.05), indicating that the procognitive effects of these compounds did not persist 48 h after their administration. However, the post hoc Tukey’s test revealed a statistically significant improvement in cognitive processes in the animals administered with xanthotoxin (15 mg/kg) and nicotine (0.1 mg/kg, s.c.), as compared with the mice treated only with xanthotoxin or nicotine (*p* < 0.05, *p* < 0.01, respectively).

For memory consolidation during the retention trial, two-way ANOVA revealed a statistically significant effect caused by the administration of xanthotoxin (15 mg/kg) and nicotine (0.1 mg/kg) (pretreatment [F(1,28)=5.71, *p* = 0.0239], treatment [F(1,28) = 24.39, *p* < 0.0001], and interaction effects [F(1,28) = 11.24, *p* = 0.0023]). The post hoc Tukey’s test showed that neither xanthotoxin nor nicotine induced significant changes in the IL value as compared with the saline-treated mice (*p* > 0.05). However, the post hoc Tukey’s test revealed a statistically significant effect in memory and learning processes in the animals administered with xanthotoxin (15 mg/kg) and nicotine (0.1 mg/kg) in combination as compared with mice treated with only xanthotoxin or nicotine (*p* < 0.001) 48 h after injection (Fig. 3b (a)).

Figure 3a (b) indicates the effects of the injection of bergapten (25 mg/kg) and nicotine (0.1 mg/kg, s.c.), alone or in combination on memory acquisition 48 h after drug administration in the PA task (two-way ANOVA: interactions [F(1,25) = 4.41, *p* = 0.046] without pretreatment [F(1,25) = 2.31, *p* = 0.1410] and treatment effects [F(1,25) = 0.22, *p* = 0.6458]). In the bergapten- and nicotine-treated groups, there were no significant changes in the IL value as compared with the saline-treated mice (*p* > 0.05), indicating that these drugs did not influence the acquisition of memory and learning 48 h after their injection. However, the post hoc Tukey’s test revealed a statistically significant improvement in cognitive processes in the animals administered with bergapten (25 mg/kg) and nicotine (0.1 mg/kg) as compared with the nicotine-treated mice (*p* < 0.05).

For memory consolidation during the retention trial, two-way ANOVA did not reveal any statistically significant effect caused by administration of bergapten and nicotine (pretreatment [F(1,30) = 0.33, *p* = 0.5706], treatment [F(1,30) = 2.57, *p* = 0.1197], and interaction effects [F(1,30) = 0.90, *p* = 0. 9324]). The post hoc Tukey’s test showed that neither bergapten nor nicotine induced significant changes in the IL value as compared with the saline-treated mice (*p* > 0.05). Also, the post hoc Tukey’s test did not reveal any statistically significant effect in memory and learning processes in the animals administered with bergapten (15 mg/kg) and nicotine (0.1 mg/kg) 48 h after injection (Fig. 3b (b)).

Figure 3a (c) indicates the effects of the injection of umbelliferone (25 mg/kg) and nicotine (0.1 mg/kg, s.c.), alone or in combination, on memory acquisition 48 h after drug administration in the PA task (two-way ANOVA: pretreatment [F(1,27) = 4.43, *p* = 0.0008], treatment [F(1,27) = 6.81, *p* = 0.0146], and interaction effects [F(1,27) = 6.12, *p* = 0.0190]). In the umbelliferone- and nicotine-treated groups, there were no significant changes in the IL value as compared with the saline-treated mice (*p* > 0.05), indicating that these drugs did not influence the acquisition of memory and learning 48 h after injection. On the other hand, the post hoc Tukey’s test revealed a statistically significant improvement in the cognitive processes in those animals administered with umbelliferone (25 mg/kg) and nicotine (0.1 mg/kg, s.c.), as compared with the nicotine-treated mice (*p* < 0.001) and umbelliferone-treated groups (*p* < 0.01).

For memory consolidation during the retention trial, two-way ANOVA did not reveal any statistically significant effect caused by the administration of umbelliferone and nicotine (treatment [F(1,25) = 5.45, *p* = 0.0278], without pretreatment [F(1,25) = 2.62, *p* = 0.1182], and interaction effects [F(1,25) = 0.01, *p* = 0.9370]). The post hoc Tukey’s test showed that neither umbelliferone nor nicotine induced significant changes in the IL value as compared with the saline-treated mice (*p* > 0.05). Also, the post hoc Tukey’s test did not reveal any statistically significant effect in memory and learning processes in the animals administered with umbelliferone (15 mg/kg) and nicotine (0.1 mg/kg) 48 h after injection (Fig. 3b (c)).

### Effects of xanthotoxin, bergapten, and umbelliferone on depression-like behaviors induced by nicotine in the FST 48 h after drug administration

**Fig. 4:**
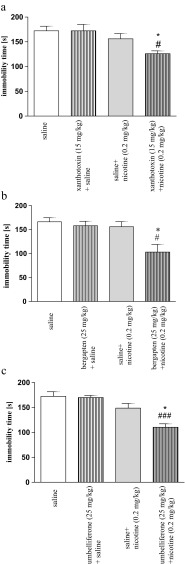
Effects of the injection of xanthotoxin (15 mg/kg; **a**), bergapten (25 mg/kg; **b**), and umbelliferone (25 mg/kg; **c**) on nicotine-induced (0.2 mg/kg) immobility time after extinction using the FST in mice. The test was performed 48 h after drug administration. Data represent the means ± SEM; *n* = 8–10; *p <0.05 vs. nicotine-treated control group; #*p* < 0.05, ###*p* < 0.001; vs. the selected coumarin (xanthotoxin, bergapten, or umbelliferone)-treated control group; Tukey’s test

In the group injected with xanthotoxin and nicotine, two-way ANOVA revealed a significant treatment effect [F(1,28) = 9.14, *p* = 0.0053], without interaction [F(1,28) = 2.11, *p* = 0.1579] and pretreatment effects [F(1,28) = 2.08, *p* = 0.1606]. In the xanthotoxin- and nicotine-treated groups, there were no significant changes in the immobility time as compared with the saline-treated mice (*p* > 0.05), indicating that these drugs did not influence the depression-like behavior 48 h after injection. Coadministration of xanthotoxin (15 mg/kg) and nicotine (0.2 mg/kg) decreased the immobility time in the FST vs. the xanthotoxin- or nicotine-treated mice (*p* < 0.05, post hoc Tukey’s test) (Fig. 4a).

In the group injected with bergapten and nicotine, two-way ANOVA revealed a significant treatment effect [F(1,32) = 8.47, *p* = 0.0065] and pretreatment effect [F(1,32) = 7.41, *p* = 0.0104] without interactions [F(1,32) = 4.00, *p* = 0.0539]. In the bergapten- and nicotine-treated groups, there were no significant changes in the immobility time as compared with the saline-treated mice (*p* > 0.05), indicating that these drugs did not influence the depression-like behavior 48 h after injection. Coadministration of bergapten (25 mg/kg) and nicotine (0.2 mg/kg) decreased the immobility time in the FST vs. the bergapten- or nicotine-treated mice (*p* < 0.05, post hoc Tukey’s test) (Fig. 3b).

In the group injected with umbelliferone and nicotine, two-way ANOVA revealed a significant treatment [F(1,27) = 24.94, *p* < 0.0001], pretreatment [F(1,27) = 5.98, *p* = 0.0213], and interaction effects [F(1,27) = 4.76, *p* = 0.0380]. In the umbelliferone- and nicotine-treated groups, there were no significant changes in the immobility time as compared with the saline-treated mice (*p* > 0.05), indicating that these drugs did not influence the depression-like behavior 48 h after injection. Coadministration of umbelliferone and nicotine decreased the immobility time in the FST vs. the umbelliferone- or nicotine-treated mice (*p* < 0.001, *p* < 0.05, respectively, post hoc Tukey’s test) (Fig. 4c).

### Effects of coumarins and nicotine on locomotor activity

Neither coumarins nor nicotine (0.1 and 0.2 mg/kg) administered separately influenced locomotor activity of mice 60 min after administration (mean photocell beam-break: saline 724.61 ± 52.75; nicotine (0.1 mg/kg) 623.56 ± 117.5; nicotine (0.2 mg/kg) 651.66 ± 48.890; xanthotoxin 608.45 ± 29.72; bergapten 704.42 ± 76.28; umbelliferone 687.51 ± 49.48; one-way ANOVA F(5,52) = 0.5266, *p* = 0.0113).

## Discussion

Nicotine, the principal alkaloid of tobacco, induces diverse behavioral effects, including the procognitive (Kruk-Slomka et al. 2012; Levin et al. 2009), anxiogenic (Budzynska et al. 2013; File et al. 2000), analgesic (Marubio et al. 1999), or antidepressive activity (Hayase 2011). Additionally, smoking cessation causes somatic and affective (motivational) symptoms, which are observed within a few hours following discontinuation of nicotine intake. During nicotine withdrawal, depression is accompanied by cognitive deficits and anxiety (Hughes 2007; Stolerman and Shoaib 1991). These studies were undertaken to examine whether three natural coumarins could modulate nicotine-induced behavioral effects (memory and depression) in mice. Nicotine improved memory acquisition and consolidation measured in the PA test 24 h after drug administration (Budzynska et al. 2015). Moreover, this alkaloid showed antidepressive activity in the FST 30 min after injection and this effect persisted 24 h. Our present studies revealed that xanthotoxin, bergapten, and umbelliferone also exerted procognitive effects in PA test; whereas, they did not influence immobility time in the FST measured 24 h after drug injections. However, effects of coumarins and nicotine were completely extinguished 48 h after drug administration. Thus, for the first time, it was revealed that the two furanocoumarins, xanthotoxin, and bergapten, as well as the simple coumarin, umbelliferone, prolonged the antidepressive, as well as the procognitive effects of nicotine, and these results are not modulated by the influence of these compounds on locomotor activity.

A potential limitation of this study was that the plasma level of nicotine after coadministration of this alkaloid with coumarins was not measured, thus we cannot exclude that interactions between nicotine and coumarins may be due to pharmacodynamic interactions, not pharmacokinetic ones. However, it was revealed earlier that xanthotoxin at the dose of 15 mg/kg increased the nicotine plasma level in vivo in mice (Alsharari et al. 2014). Moreover, it was shown that xanthotoxin inhibited human CYP2A6 and CYP2A3, through different mechanisms and in a time-dependent manner (Visoni et al. 2008). It was also demonstrated that xanthotoxin inhibits cytochrome CYP2A5 and CYP2A3 in mice and rats. Human CYP2A6 is present in the liver and in the nasal mucosa of the esophagus and is involved in the biotransformation of nicotine (Visoni et al. 2008). Thus, it is concluded that the metabolism of nicotine in the mouse is very similar to that occurring in human. It was also shown that xanthotoxin significantly reduced the metabolic elimination of nicotine in vivo (Raunio et al. 2008; Sellers et al. 2003), as well as inhibited the CYP2A5-mediated metabolism of nicotine in liver microsomes (Damaj et al. 2007).

The present experiments follow the previous studies showing that pretreatment with xanthotoxin prolongs the duration of nicotine-induced antinociception and hypothermia and that this effect was associated with a prolongation of nicotine plasma levels in mice (Alsharari et al. 2014). The present results extend the findings of Bagdas et al. (2014) demonstrating that administration of xanthotoxin increases and prolongs nicotine levels thereby enhancing nicotine dependence-related behaviors in mice, indicating the relationship between the metabolism of nicotine and the nicotine addiction state in animal models (Bagdas et al. 2014). Other furanocoumarins, such as bergapten, imperatorin, and isopimpinellin, are potent inhibitors of coumarin 7-hydroxylation in mice (Chang and Waxman 1996). It was also revealed, in a study of CYP3A4 inhibition by furanocoumarins contained in grapefruit juice, that bergapten is probably the most potent inhibitor of CYP3A4, which is regarded as the major drug-metabolizing oxidase in human liver (Cuciureanu et al. 2010).

The present results demonstrated that administration of xanthotoxin, bergapten, and umbelliferone significantly prolonged nicotine-induced antidepressive effects, at a similar level. Differences between the effects of the coumarins on memory processes induced by nicotine were observed. Xanthotoxin prolonged the duration of nicotine-induced acquisition and consolidation of memory, whereas bergapten and umbelliferone influenced only memory acquisition in the PA test. These results are in agreement with previous studies from this laboratory which showed that coadministration of inactive doses of nicotine (0.05 mg/kg s.c.) and imperatorin (1 mg/kg, i.p.) provided a statistically significant improvement in acquisition of memory and learning processes and did not affect consolidation processes (Budzynska et al. 2013).

The previous study revealed that nicotine improved both memory acquisition and consolidation processes. It is established that the distinct memory stages, acquisition and consolidation are temporally and molecularly different. In these processes, mainly cholinergic, dopaminergic, and glutamatergic neurotransmissions participate, and GABA, noradrenalin, adrenaline, and serotonin are involved in memory modulation (Abel and Lattal 2001; Levin and Rezvani 2007). More recent experiments have revealed that ACh is involved mainly in the initial acquisition processes. Thus, inhibition of the muscarinic acetylcholine receptors significantly disturbs memory acquisition (Gil-Bea et al. 2011; Robinson et al. 2011). Additionally, it was shown that dopamine is more committed in the consolidation of memory processes lasting from minutes to days (Bethus et al. 2010). Nicotine, as a cholinergic receptor agonist, is known to activate the cholinergic system, and, as a drug of abuse, it also directly activates dopaminergic pathways projecting from the ventral tegmental area to the nucleus accumbens and the hippocampus (Zaniewska et al. 2009). Thus, the differences observed in the effects on memory processes among xanthotoxin, bergapten, and umbelliferone may be due to the more pronounced influence of nicotine on cholinergic transmission which were observed 48 h after drug administration, rather than on dopaminergic pathways.

The observed results may also indicate that C-8 substituted coumarins (e.g., xanthotoxin) possess stronger pharmacological activity than those with the C-5 position of the psoralen ring substituted (e.g., bergapten). This hypothesis is supported by the previous study which clearly demonstrated that imperatorin and xanthotoxin, both of which are substituted at the C-8 position of the psoralen ring, may serve as potential compounds for further development against tonic–clonic seizures. Similarly, the lack of anticonvulsant activity shown by bergapten and oxypeucedanin, which are C-5 substituted compounds, confirmed that the regio-oxygenation plays an important role in the pharmacological evaluation of natural coumarins in preclinical studies (Luszczki et al. 2010). Thus, it can be hypothesized that some modifications and/or manipulations of the chemical structures of both compounds at the C-8 position of the psoralen ring might be profitable from a preclinical development perspective. Since it was shown that the CYP2A6 gene is polymorphic, it is suggested that inhibition of this enzyme will have no negative effects. Furthermore, since not many drugs, other than nicotine, are metabolized by this enzyme, the probability of unexpected interactions would appear to be minimal (Raunio et al. 2001).

Overall, the present study showed that furanocoumarins xanthotoxin and bergapten and the simple coumarin umbelliferone prolonged the behavioral effects of nicotine. The results suggest that these metabolites, which are readily available, exert a modulating effect on the metabolism of nicotine and should be further explored as a novel pharmacotherapeutic approach for the treatment of nicotinism.
